# Optimization of a Widefield Structured Illumination Microscope for Non-Destructive Assessment and Quantification of Nuclear Features in Tumor Margins of a Primary Mouse Model of Sarcoma

**DOI:** 10.1371/journal.pone.0068868

**Published:** 2013-07-23

**Authors:** Henry L. Fu, Jenna L. Mueller, Melodi P. Javid, Jeffrey K. Mito, David G. Kirsch, Nimmi Ramanujam, J. Quincy Brown

**Affiliations:** 1 Department of Biomedical Engineering, Duke University, Durham, North Carolina, United States of America; 2 Department of Pharmacology & Cancer Biology, Duke University School of Medicine, Durham, North Carolina, United States of America; 3 Department of Radiation Oncology, Duke University School of Medicine, Durham, North Carolina, United States of America; 4 Department of Biomedical Engineering, Tulane University, New Orleans, Louisiana, United States of America; University of California, Irvine, United States of America

## Abstract

Cancer is associated with specific cellular morphological changes, such as increased nuclear size and crowding from rapidly proliferating cells. *In situ* tissue imaging using fluorescent stains may be useful for intraoperative detection of residual cancer in surgical tumor margins. We developed a widefield fluorescence structured illumination microscope (SIM) system with a single-shot FOV of 2.1×1.6 mm (3.4 mm^2^) and sub-cellular resolution (4.4 µm). The objectives of this work were to measure the relationship between illumination pattern frequency and optical sectioning strength and signal-to-noise ratio in turbid (i.e. thick) samples for selection of the optimum frequency, and to determine feasibility for detecting residual cancer on tumor resection margins, using a genetically engineered primary mouse model of sarcoma. The SIM system was tested in tissue mimicking solid phantoms with various scattering levels to determine impact of both turbidity and illumination frequency on two SIM metrics, optical section thickness and modulation depth. To demonstrate preclinical feasibility, *ex vivo* 50 µm frozen sections and fresh intact thick tissue samples excised from a primary mouse model of sarcoma were stained with acridine orange, which stains cell nuclei, skeletal muscle, and collagenous stroma. The cell nuclei were segmented using a high-pass filter algorithm, which allowed quantification of nuclear density. The results showed that the optimal illumination frequency was 31.7 µm^−1^ used in conjunction with a 4×0.1 NA objective (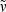 = 0.165). This yielded an optical section thickness of 128 µm and an 8.9×contrast enhancement over uniform illumination. We successfully demonstrated the ability to resolve cell nuclei *in situ* achieved via SIM, which allowed segmentation of nuclei from heterogeneous tissues in the presence of considerable background fluorescence. Specifically, we demonstrate that optical sectioning of fresh intact thick tissues performed equivalently in regards to nuclear density quantification, to physical frozen sectioning and standard microscopy.

## Introduction

Fluorescence microscopy is an attractive optical technique capable of generating high- resolution images for visualizing tissue microstructure. One significant morphological change that occurs in cancer is enlargement of nuclear size and increase in nuclear density (i.e. reduction in average inter-nuclear distance) [1]. This is a characteristic, which is commonly exploited by pathologists, to diagnose cancer pathology in hematoxylin and eosin (H&E) stained tissues sections. An appropriate contrast agent could be used with fluorescence microscopy to visualize cell nuclei without requiring extensive sample preparation. This provides an advantage over H&E as the tissue can be imaged intra-operatively *in vivo* or *ex vivo* without the need for fixation or H&E staining. Several groups have demonstrated the use of high resolution imaging in combination with nuclear-specific contrast agents to visualize morphological changes in cancer. Gareau et al. have demonstrated in several publications the use of confocal microscopy to image skin tumors stained with acridine orange (AO) stain [2], [3]. Richards-Kortum et al. have imaged dysplasia and early cancer in the oral cavity and esophagus stained with acriflavine using a high resolution fluorescence microendoscope [1], [4], [5]. Gmitro et al. have also demonstrated imaging of ovarian tissue and cancer stained with AO using confocal scanning microscopy[6]–[9].

A number of clinical applications exist which require direct imaging at the microscopic level, specifically in the realm of cancer diagnosis. One particular clinical need is in assessing the margin of a tumor once it has been surgically removed from a patient. Tumor excision is a very common treatment in solid cancers, where the surgeon must remove all of the diseased tissue from the patient to minimize the risk of cancer recurrence. Currently there are very few intra-operative tools available to assist surgeons in tumor margin diagnosis to ensure complete removal of the tumor. As an example, in breast cancer, over 20–70% of patients must undergo a secondary re-excision after the initial surgery due to a positive margin on the excised specimen in post-operative H&E histopathology [10]–[15]. Touch prep cytology and frozen section analysis are two intra-operative tools currently implemented in some clinics, but both require a trained pathologist and other resources [16], [17]. Optical imaging techniques are particularly attractive for this application as entire margins can be intra-operatively imaged non-destructively and potentially, *in situ*. Many recent studies have reported the utility of various optical modalities as possible approaches to diagnosing surgical tumor margins [18], [19]. Our group has developed instrumentation for widefield tissue diffuse reflectance spectral imaging, to exploit intrinsic absorption and scattering contrast in breast cancer tumor margins [19]. Additionally, other groups have developed similar approaches and presented alterative designs for spectral imaging systems which generate optical absorption and scattering maps of tumor margins [20]–[23]. Another optically based approach for identifying tumor margins is through the use of specifically targeted contrast agents. Several groups have demonstrated using these contrast agents for demarcating various tumors tissues for fluorescence guided resection [24]–[27]. While it is clear that many optical approaches exist to address margin assessment, the focus of the work presented in this publication specifically focused on optical assessment of the tumor margin at the microscopic level. Nguyen et al. [28] demonstrated imaging breast tumor samples using optical coherence tomography to visualize scattering contrast at microscopic resolutions (35 µm lateral and 5.9 µm axial) in breast tumor margins. An alternative approach, which leverages similar spatial resolution and sources of contrast as traditional histopathology, is to use fluorescence microscopy combined with a suitable fluorescent stain applied to the fresh, intact surgical specimen. This allows for the assessment of tumor markers, for instance changes in nuclear size, shape, and inter-nuclear distance, which are commonly used in histopathologic assessment.”

In conventional fluorescence microscopy, the entire field of view is uniformly illuminated with the excitation light. This creates a problem as fluorophores outside the plane of focus are also excited and emit photons, generating a significant amount of unwanted background fluorescence. This in turn significantly degrades contrast of features of interest in the focal plane. This is a common issue in widefield fluorescence microscopy and several specialized techniques exist to reject background fluorescence, such as fluorescence confocal microscopy. In fact, Gareau et al. recently reported a custom fluorescence confocal microscopy system designed to image and diagnose margins during Mohs procedures for removal of skin cancer [29]. While extremely effective in rejecting background fluorescence and capable of exquisite image quality, confocal microscopy typically relies on sequential pixel scanning, in which a laser beam is focused to a diffraction-limited spot and scanned over the sample in time. Confocal scanning approaches which aim to parallelize the acquisition process, such as spinning disk confocal and line-scan confocal, serve to increase the effective pixel sampling rate, but not without limitations (spinning disk confocal is limited in the adjustment of axial resolution, and line scan confocal with linear CCDs results in possibly asymmetric lateral resolution along the line dimension).

An alternative approach to reject background fluorescence with fully parallel pixel detection is structured illumination microscopy (SIM), in which the entire field of view is illuminated with a defined spatial pattern and scanning of a focal spot is not required [30]. Other than the use of patterned illumination, the illumination and collection geometry is identical to that of conventional widefield fluorescence microscopy, so a standard CCD may be used for detection. The advantages of structured illumination microscopy are that 1) optically-sectioned images are obtained with all pixels in parallel, thereby significantly increasing the pixel sampling rates, 2) CCD detectors with high quantum efficiency can be used making SIM light-efficient, 3) the axial resolution can be tuned by varying pattern frequency, and the lateral resolution is symmetric over the field of view, and 4) it is a relatively low complexity solution with no moving parts. Structured illumination microscopy has been shown to perform equivalently (and at times better) than confocal microscopy with respect to optical sectioning and SNR, particularly in superficial tissues [31]–[33]. Disadvantages of structured illumination microscopy include 1) the amplification of shot noise from the out-of-focus background, 2) the reduction of recovered signal when the illumination frequency is near the cut-off frequency of the imaging optics [34], which can reduce the image quality compared to confocal, and 3) its reduced performance in deep imaging as compared to confocal [31]. However the advantages of structured illumination microscopy in terms of imaging throughput and reduced complexity may outweigh the disadvantages in imaging performance when the superficial surface of large areas of tissue are to be scanned (average breast margin size, 20 cm^2^ [19]).

While structured illumination microscopy has been applied to a wide range of samples, the majority of previous publications have focused on imaging of cells in culture or optically clear samples [30], [33], [35]–[37]. A few groups have successfully applied this technique on thick biological tissues. Elson et al. [38] demonstrate the effect of optical sectioning in thin slices of mouse ear. Santos et al. [39] used HiLo microscopy, a specialized form of structured illumination for optical sectioning, through a fiber bundle microendoscope to image rat mucosa *ex vivo*. More recently, Lim et al. [40] has also utilized HiLo microscopy in the hippocampus region of *ex vivo* rat brain. There have been limited reports of applying structured illumination to thick, intact, highly scattering biological samples. Mazher et al. [41] have previously demonstrated structured illumination imaging in a thick tissue-mimicking phantom. However, their implementation and analysis focused on the diffusion regime (mm to cm spatial scales), rather than the diffraction regime, which is more relevant to our microscopic imaging application.

The optical section thickness is dependent on frequency selected for illumination. Chasles et al. [31] provided an in-depth theoretical and experimental analysis of the effect of different grid frequencies on axial resolution and compared structured illumination microscopy directly to other optical sectioning techniques. They determined that the axial resolution was improved by a factor of 1.5 when using structured illumination over conventional widefield microscopy. Karadaglic et al. [42] presented a detailed theoretical analysis showing that the optical sectioning thickness could be appropriately estimated using the Stokseth approximation [43] of the optical transfer function of a defocussed imaging system. However, this derivation was calculated assuming the sample was a thin fluorescent sheet scanned axially in the absence of turbidity. The exact effect of a scattering background on the optical section thickness has not been fully explored. In addition, the modulation depth, which is a measurement of the transfer of the illumination pattern contrast to the focal plane, is also directly related to the illumination frequency and scattering of the sample. Understanding this relationship is vital as the modulation depth has an impact on the signal to noise ratio (SNR) of the sectioned image [32] and the optimal illumination frequency has to balance a tradeoff between optical section thickness (which is better at higher frequencies) vs. modulation depth and SNR which degrades as frequency increases.

The primary goal of this study was to design and optimize a SIM imaging system with the requisite parameters to image tumor margins. Specifically, these included the ability to image fresh tissue immediately after removal from the subject, with sufficient lateral resolution and optical section thickness that effectively reduces out-of-focus fluorescence to isolate single nuclei, and a field of view which may be reasonably scaled to cover a large tumor margin. Optical section thickness and modulation depth were measured over a range of illumination frequencies in specially-designed tissue simulating phantoms of varying turbidity levels. Specific illumination frequencies suitable for fluorescence microscopy of thick tissues were determined based on an analysis of optical sectioning strength, modulation depth and SNR using the phantoms. The system was then tested on tissue from a genetically engineered primary mouse model of sarcoma to demonstrate biological feasibility in identifying tumor margins. Frozen tissue sections (∼50 µm in thickness) were used to demonstrate the morphological congruence between SIM microscopy and the corresponding H&E-stained section. Freshly excised intact thick tissues (tumor, muscle and combinations of tumor and muscle) were imaged *in situ* to demonstrate the optical sectioning capability of the system. Finally a high-pass filter algorithm was applied to the sectioned images acquired from the intact tissue and to uniform images acquired from the frozen sections, to compare the performance of the optical sectioning system versus physical tissue sectioning for quantifying nuclear density.

## Methods

### Ethics Statement

This study was carried out in strict accordance with the recommendations in the Guide for the Care and Use of Laboratory Animals of the National Institutes of Health. The protocol was approved by the Duke University Institutional Animal Care and Use Committee (Protocol Number: A134-10-05). All surgery was performed under isoflurane gas anesthesia, and all efforts were made to minimize suffering.

### Structured Illumination Theory

The implementation of SI involves illuminating a sample using a sinusoidal pattern defined by the following equation:
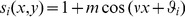
(1)


The quantity *m* represents the modulation depth (value between 0 and 1), *ν* represents the spatial frequency, and *φ_i_* represents the phase shift of the pattern. The measured image intensity of a sample illuminated by this pattern can be described by the following:
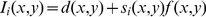
(2)


In this equation, *d(x,y)* represents the fluorescence emitted from the sample which is out of focus, and *f(x,y)* represents the in-focus fluorescence. As only the in-focus fluorescence from the object is modulated by the sinusoidal component of the illumination pattern, a proper demodulation method can be applied to extract only this information. The most straightforward and commonly used algorithm relies on square law detection to extract and demodulate the in-focus component.
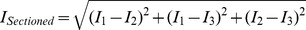
(3)


As the equation shows, the image, which contains only information from the focal plane, *I_Sectioned_*, is calculated by acquiring three separate images (*I_1_, I_2_, I_3_*) which differ only in phase shift (*φ_1_* = 0, *φ_2_* = 2π/3, *φ_3_* = 4π/3). The resulting sectioned image thus only contains the modulated portion of the object, which corresponds to the plane of focus.

### System Design

In order to achieve comparable performance to the current histopathologic methods, the structured illumination microscopy (SIM) system was designed to resolve cell nuclei. The cell nuclei diameter seen in our intended pre-clinical model, a mouse sarcoma tumor, is in the range of 5–15 µm [44]. Furthermore, human breast cancer nuclei sizes are at least 8 µm in diameter [45]. The field of view (FOV) was also an important consideration which was determined by both the size of the sample and desired imaging time. Because of the relatively large sizes of the preclinical mouse sarcoma margins (15 mm^2^) and breast tumor margins (20 cm^2^ [46]), the system was designed with the largest per-frame FOV as possible, while maintaining the required lateral resolution which in our case was 2.5 mm^2^ (1.58 × 1.58 mm). AO was chosen as the contrast agent in this study because it has been demonstrated to stain nuclei, skeletal muscle, and collagenous stroma [2], [7]. While our studies involved imaging samples *ex vivo*, it is worth noting that AO has been approved for use in humans in at least one previous study [7]. As an alternative, other non-propidium iodide nuclear markers, such as proflavine and acriflavine, have similar staining properties and proflavine, in particular, has been deemed safe for human use and is used as a disinfectant for the umbilicus.

**Figure 1:**
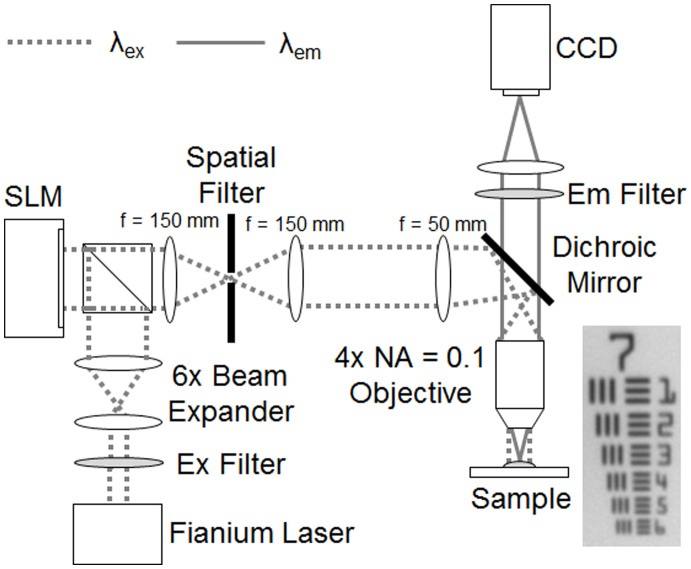
Detailed schematic of the SIM imaging system. The λ_ex_ peak was 480 nm and the λ_em_ peak was 520 nm. The spatial filter diameter was adjusted to allow only the 0 and +1 diffraction orders to pass. An image of group 7 of the USAF resolution target acquired by the system is also shown.

A detailed schematic of the system is shown in Figure 1. A broadband super continuum laser (Fianium SC400) was used to provide illumination for fluorophore excitation. This source was chosen due to its low coherence which mitigated the appearance of speckle in the acquired images. The output from the laser traveled through a band-pass filter centered at 480 nm with a bandpass of 20 nm, which corresponded to the excitation peak of AO. This filter can potentially be replaced to match the excitation peak of other intra-vital dyes or even intrinsic fluorophores. The filtered beam was then passed through a 6×beam expander and a polarizing beam splitter that redirected the light toward an LCoS SLM display chip (Holoeye LCR-720). Following reflection off the SLM chip, the light traveled through a series of 4 lenses, the last of which was the microscope objective (Nikon 4×E Plan Fluor, NA = 0.1). An iris placed one focal length after the first lens was used to spatially filter the diffraction orders created by the sinusoidal pattern on the SLM. The iris was aligned on the optical axis to only pass the 0 and +1 diffraction orders. Allowing these two diffraction orders to pass yielded a sinusoidal pattern at the sample plane, free of undesired higher frequency harmonics, which produce sectioning artifacts if allowed to pass to the sample. The resulting fluorescence generated by the illumination pattern incident on the sample was collected by the objective and imaged onto the CCD (LaVision Imager 3 QE) using a 200 mm focal length tube lens (Nikon MXA20696).

### System Imaging Parameters Characterization

Once the system was constructed according to the design specifications above, the basic imaging parameters of the microscope were characterized. For a Nikon 4×objective with numerical aperture (NA) of 0.1, the diffraction limited lateral resolution is expected to exceed the value needed for visualization of cell nuclei (3.2 µm, based on the Rayleigh criterion calculation and emission peak of AO). The actual lateral resolution of the system was measured using a standard 1951 USAF Resolution test target. The test target was placed at the focal plane of the objective with a fluorescence calibration test slide underneath (outside of the focal plane). Uniform excitation light was projected onto the sample and fluorescence emitted from a calibration slide through the test target, which was imaged onto the CCD. The smallest resolvable line pair group was deemed as the measured lateral resolution of the SIM system. In addition, the single frame field of view (FOV) was also measured using a larger line pair group from the same test target.

Because of the finite pixel number and size of the SLM, a limited number of discrete frequencies could be produced for structured illumination. Using the experimentally measured FOV, the value of each discrete spatial frequency (in mm^−1^) at the sample plane (for each pattern generated by the SLM) was determined by imaging the fluorescence from a uniform calibration test slide and calculating the Fourier Transform of the resulting image.

### Tissue Phantom Preparation

A set of phantoms was constructed in order to simulate the type of environment seen in thick tissue samples stained with AO. Each phantom consisted of fluorescence spheres (Polysciences, Fluoresbrite YG Microspheres) and TiO_2_ (Sigma, T8141) in a polydimethylsiloxane (PDMS) sample (Dow Corning, Slygard 184). The phantoms were constructed in a petri dish with a cover glass window on the bottom (Mattek, P35G-0-14-C). The first set of phantoms used a layer of 1-µm diameter fluorescent spheres dried on the cover glass to generate an optically thin layer of fluorescence (simulating the superficial layer of AO in tissue). A 1 cm layer of PDMS was added behind the fluorescent layer with variable concentrations of TiO_2_ to create three separate phantoms, each with different scattering levels where the reduced scattering coefficients were µ_s_′ = 0 cm^−1^, 10 cm^−1^, and 20 cm^−1^. A quantity of 0, 2.25, and 4.50 grams of TiO_2_ per gram of uncured PDMS was added to the PDMS for each respective scattering level, calculated according to a previously published procedure [47]. The TiO_2_ was thoroughly mixed with the PDMS prior to curing. Finally the phantom was placed in a vacuum chamber to draw out all residual air bubbles from the mixing process and also to effectively cure the PDMS.

Another solid phantom was constructed using PDMS, 10-µm diameter fluorescent polystyrene spheres and TiO_2_ spheres. The size of the fluorescent spheres was chosen to simulate the size of targets the system was designed to detect (cell nuclei). The underlying PDMS and TiO_2_ layer was mixed to generate a single scattering coefficient of 10 cm^−1^, which is a commonly measured reduced scattering coefficient in soft tissues [19], [46].

### Structured Illumination Characterization

The phantom consisting of a single layer of 10-µm spheres with a background reduced scattering coefficient of 10 cm^−1^ was used to demonstrate the contrast improvement by computing the signal to background in the structured illumination (i.e. sectioned) and uniform illumination (i.e., non-sectioned) images. The contrast ratio was quantified by taking the mean fluorescence intensity of a sphere and dividing it by the mean intensity of the adjacent background. Additionally, the 10-µm spheres were representative of typical size cell nuclei both in mouse and human tissue, so this phantom was an appropriate biological model for testing the system.

**Figure 2:**
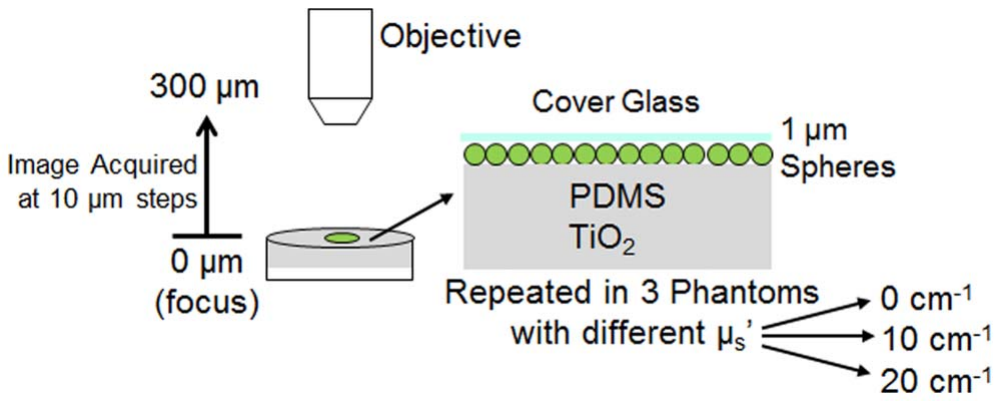
Schematic demonstrating the method used to measure the optical sectioning strength of the imaging system. Also shown is a detailed diagram of the structure of the solid phantom used for measurement. Three separate phantoms were creating with increasing levels, µ_s_′ = 0, 10, 20 cm^−1^.

A straightforward procedure was used to experimentally determine the effective optical section thickness of the SIM system, as illustrated in Figure 2. The thin fluorescent phantoms (single layer of 1 µm spheres) were translated axially toward the objective and a sectioned image (using structured illumination) was acquired at each 10 µm step. The mean fluorescence intensity from a region of interest (ROI) within the sectioned image was plotted against axial depth to determine the section thickness of the system. In addition, the section thickness was measured over a range of illumination pattern frequencies, for each of a range of phantoms with different scattering properties.

In order to verify the experimental optical section thickness measurements, the results were compared to predicted theoretical results. The defocus of the structured illumination pattern has been previously shown to match the Stokseth empirical approximation of the optical transfer function [42], [43].
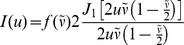
(4)where 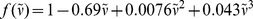




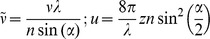
Where *I(u)* is the optical sectioning axial response,*J_1_* is the Bessel function of the first kind, *ν* is the grid frequency (as shown in Eq. 1), 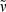 is the normalized grid frequency, *λ* is the emission wavelength, *nsin(α)* is the NA of the objective, *z* is the real axial distance, and *u* is the normalized axial distance. The Stokseth approximation provided a valid theoretical optical section thickness value to verify measured results.

Identifying the exact relationship between section thickness and illumination frequency was only the first step in selecting the appropriate frequency for imaging. The modulation depth, *m* in the previously shown Eq. 1, is a quantity, which represents the amplitude of the sinusoidal illumination pattern transferred onto the sample. The importance of the modulation depth becomes apparent after combining Eq. 1 and Eq. 2 with Eq. 3 which results in the following [32]:
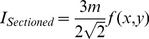
(5)


As expected, we can see that the resulting signal of the sectioned image, *I_Sectioned_*, is described primarily by the in-focus fluorescence, *f(x,y)*. However, a coefficient dependent on modulation depth, *m*, appears in this equation and scales *f(x,y)* accordingly. In addition, modulation depth decreases with frequency because of the natural decay in the shape of the incoherent optical transfer function of the objective. The following procedure detailed by Hagen et al. [32] was used to measure the modulation depth using the three phase shifted structured illumination images (I_1_, I_2_, I_3_):
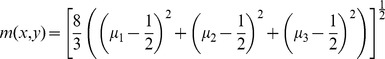
(6)where 
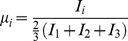


This method can be used to measure the modulation depth, *m(x,y)* at each pixel within a given image. The median value was chosen to represent the modulation depth over a specific ROI in each image, where fluorescent targets were present. This median modulation depth was measured for a range of frequencies (using the same ROI and sample location) on the same fluorescent phantoms used to characterize the optical section thickness. The phantom was simply placed in focus at the sample plane and three phase shifted images at multiple illumination frequencies were acquired.

Because of the direct relationship of modulation depth on recovered signal, and the inverse relationship between SNR and modulation depth, an illumination frequency yielding both a sufficient modulation depth and optical section thickness must be selected. The SNR of a sectioned image is a quantitative measurement of image quality, which is directly influenced by modulation depth. Using the derivations of Hagen et al. [32], the following equations were used to calculate the SNR of the uniform and sectioned images respectively:
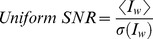
(7)

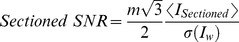
(8)


where 
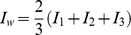


Here, I_W_ represents the traditional widefield image that is reconstructed from the three phase shifted structured illumination images. The sectioned image, I_Sectioned_, is calculated using Eq. 4. The modulation depth (introduced in Eq. 1), *m*, has a direct impact on the SNR of the sectioned image as shown in Eq. 8. Thus, the measurements of optical section thickness, modulation depth and corresponding SNR characterized at multiple frequencies, were used to provide insight into selecting a single illumination frequency that provided the best trade-off between these parameters.

### Imaging of Tumor Margins in a Primary Mouse Model of Soft Tissue Sarcoma

In order to demonstrate feasibility for imaging tumor margins, the system was tested in a primary mouse model of sarcoma. This model, which was also used for biological verification in previous margin imaging studies [26], provides a controlled environment of spontaneous tumor growth, and serves as an appropriate preclinical test bed for our system. Primary sarcomas were induced by intramuscular injection of mice with conditional mutations in oncogenic K-ras or Braf and p53 with an adenovirus expressing Cre recombinase as previously described [44], [48]. The tumor was grown to approximately 500–700 mm^3^ and then surgically removed from the animal, then two separate imaging protocols were followed. The first was to establish congruence between the morphology imaged by the SIM system and histopathology by imaging frozen sections. The second was to establish the feasibility of using SIM microscopy to image margins by examining freshly excised sarcoma tissues.

Frozen sections were used to demonstrate that the fluorescent staining approach was capable of highlighting tissue morphology comparable to traditional H&E stained histology slides. Immediately after removal from the mouse, the excised sarcoma tissue was embedded in optimal cutting temperature compound (OTC) and flash frozen in liquid nitrogen. The frozen tissue block was sliced into 50 µm thick sections spaced 50 µm apart and placed on a microscope slide. To prepare the sample for SIM imaging, the tissue sections were allowed to thaw (∼5 minutes) and a solution of 0.01% AO (mixed with water) was applied to stain the tissue. The stained tissue sections were then imaged using the SIM system and then immediately fixed in formalin. Next, the fixed tissue sections were stained with H&E and imaged using a standard bright-field microscope.

For the second protocol, the fresh, intact tumor specimen was stained with a 0.02% solution of AO immediately after tumor excision. After ∼30 seconds, the tissue was thoroughly rinsed with a phosphate-buffered saline solution to remove any excess contrast agent. A coverglass was placed over the stained tumor tissue to create a flat surface and images were acquired using the SIM system. This procedure was repeated when imaging normal skeletal muscle tissue excised from the normal contralateral hind limb of the mouse. Multiple sites were imaged from a single mouse to acquire a sufficient image dataset for this protocol.

### Quantitative Image Processing

As previously mentioned, a well-known hallmark of cancer is increased cell nuclear density, due to the increased rate of growth in malignant tissue. To exploit this information, a high pass filter (HPF) algorithm was applied to segment and isolate the cell nuclei from other features within the image. Specifically, a Gaussian filter with a standard deviation of 20 pixels was convolved with each maximum-intensity normalized image (implemented using MATLAB). The standard deviation was empirically chosen such that a majority of the nuclei were isolated with HPF. Each pixel corresponded to 1.5 µm given the combination of the specific CCD used for detection and 4×magnification objective for imaging. The typical diameter of a cell nucleus is 5–15 µm, which corresponded to approximately 3–10 pixels. Then, a threshold value of 0.1 (10% of the peak intensity) was applied to the output from the HPF, and any pixel that was greater than 0.1 was considered part of a nucleus. Next, a connected components algorithm, in which connected pixels are assumed to belong to the same cell nucleus, was applied to extract the number of cell nuclei from the filtered image.

The quantitative image processing algorithm was first applied to the images acquired in frozen tissue sections. The goal was to determine the expected nuclear density observed in sarcoma tumor tissue. The frozen tissue sections were well-suited for this purpose as they had corresponding and congruent H&E sections, which are widely used by pathologists for diagnosis. Two frozen sections from four different mice (N = 4) were acquired and processed using the HPF algorithm to isolate and count the number of cell nuclei per mm^2^. Because of differing tumor sizes, an ROI (350×350 pixels) of solid tumor was manually selected in each image. The average density was calculated over all four mice, which was then used as a benchmark when imaging thick excised sarcoma tumor margins.

However, in contrast to the frozen section samples, background rejection using SIM is vital in thick sarcoma margins to reject background fluorescence and the illumination frequency is an important factor in determining the tradeoff between optical section thickness and modulation depth. Multiple sites (N = 5) from a freshly excised intact sarcoma tumor harvested from one mouse was imaged at multiple frequencies. The extracted nuclear density from the optically sectioned thick tissue images using the HPF algorithm was calculated over a manually selected ROI the same size (350×350 pixels) used in the frozen section analysis. The average nuclear density at each illumination frequency was compared to the benchmark nuclear density established from frozen section imaging using a Wilcoxon rank-sum test. For all tests, a p value of less than 0.05 was considered to reject the null hypothesis. Measuring and understanding this information provided further justification for selection of a specific illumination frequency to optimize the extraction of nuclear density in the tumor margin of the genetically engineered mouse model of sarcoma.

## Results

### Characterization of Imaging Parameters

The single frame field of view (FOV) and lateral resolution were the first imaging parameters measured using a 1951 USAF resolution target. Using a 4×objective, the smallest element on the target (group 7, element 6) corresponding to a lateral resolution of 4.4 µm, was clearly resolved, with a single frame field of view (FOV) of 2.06×1.56 mm (3.21 mm^2^). This measured resolution met our previously mentioned design criteria both in terms of enabling visualization of individual cell nuclei (in both preclinical mouse models and human tissue) as well as the single-frame FOV to allow imaging of typically sized sarcoma tumor margins.

Table 1 shows a complete list of all illumination frequencies that were achieved at the sample plane, with the corresponding objective lens. The 4×objective was used in the phantom and sarcoma margin imaging studies due to the wider range of higher illumination frequencies at which the samples could be imaged.

**Table 1:** List of illumination frequencies achieved at the sample plane for two different objective lenses.

Objective	Absolute Frequency at Sample Plane (mm^−1^)
4×(NA = 0.1)	101, 67.0, 50.3, 47.7, 40.7, 31.7, 24.1, 19.6
2×(NA = 0.1)	25.1, 16.7, 12.5, 10.2

### Characterization of Structured Illumination in Phantoms

**Figure 3:**
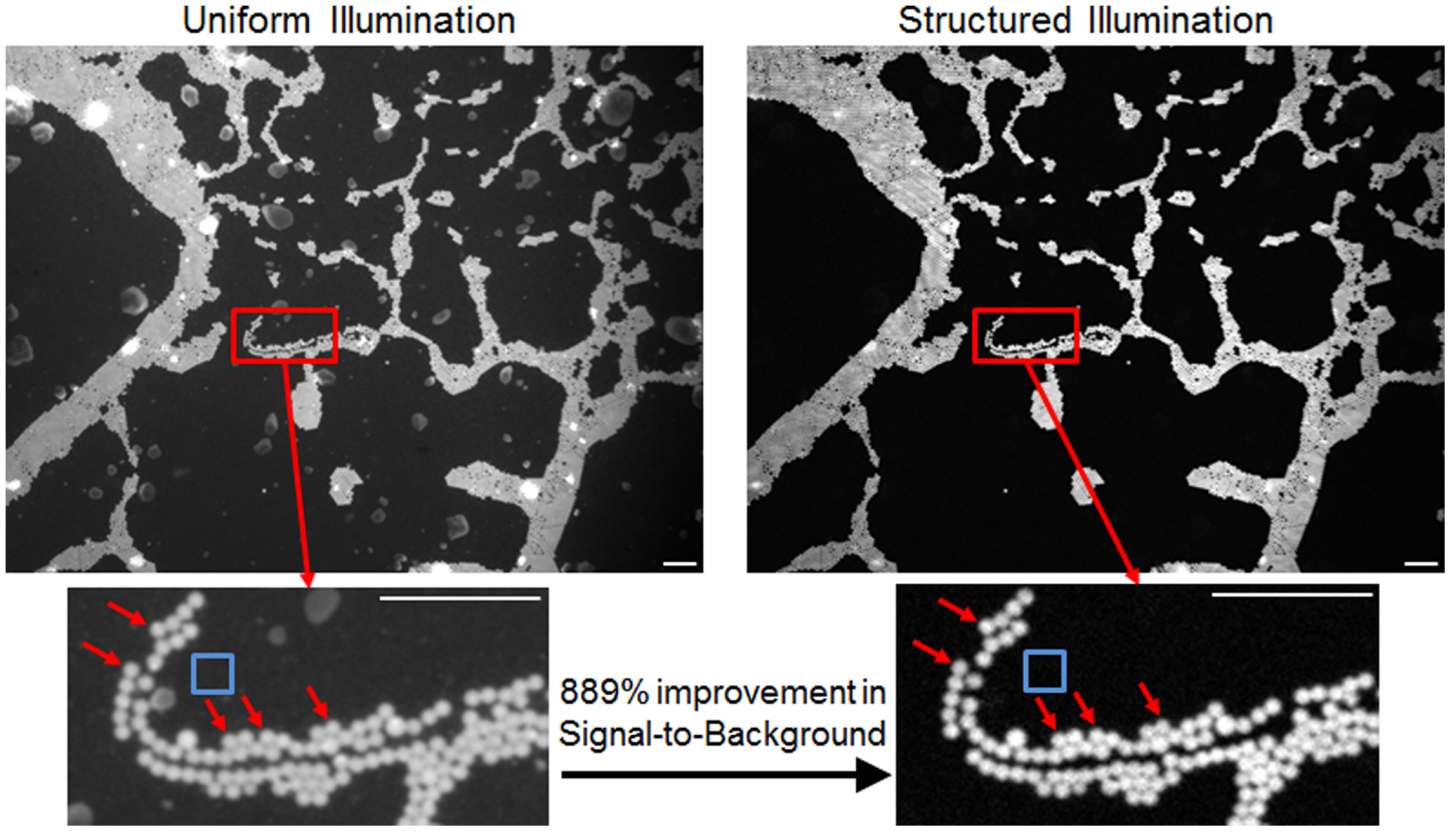
Images of a single layer of 10 µm fluorescent spheres embedded in PDMS and TiO_2_. The reduced scattering coefficient of the phantom is approximately 10 cm^−1^. Images were taking using a 4×NA = 0.1 objective and an illumination frequency of 31.7 mm^−1^. The improvement in contrast is clearly seen from the uniform to structured illumination image. The signal-to-background was calculated by taking the intensity of 5 manually selected spheres (indicated by red arrows) and dividing by the background ROI (indicated by the blue square). All scale bars are 100 µm.

Images were first taken on the phantoms consisting of single layer of 10-µm diameter fluorescence spheres. The spheres were embedded in PDMS and Ti0_2_ giving the surrounding medium a biologically relevant reduced scattering coefficient value (µ_s_′ = 10 cm^−1^) to introduce background fluorescence. The resulting images are shown in Figure 3, under both uniform illumination and structured illumination. Each image was normalized by dividing each pixel by the maximum intensity within the image. The images clearly demonstrate the in improvement seen in structured illumination compared to uniform illumination. The specific illumination frequency chosen to acquire these images was 31.7 µm. The contrast ratio was calculated directly in both images by manually selecting ROIs for 5 spheres and dividing by a background ROI (both indicated in Figure 3). The contrast ratio was averaged over these five spheres, which showed a significant quantitative improvement, 889±58% greater in the sectioned image over the uniform image.

**Figure 4:**
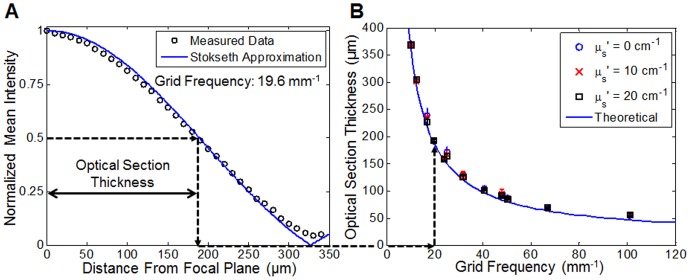
Optical section thickness measurements from solid phantoms. (**A**) Plot of the mean image intensity as a function of distance from focal plane used to determine optical section thickness. The circles represent data that was acquired on the µ_s_′ = 0 cm^−1^ phantom using a 4×NA = 0.1 objective with an illumination frequency of 19.6 mm^−1^. The solid line represents the Stokseth approximation (Eq. 4) calculated using the same variables. (**B**) Plot relating optical section thickness to illumination grid frequency for a range of reduced scattering coefficients. The datapoints represent the measured values on phantoms, and the solid line represent the theoretical value calculated using the Stokseth approximation. The dotted arrows show how the optical section thickness was measured on the left and how it was placed on the corresponding plot on the right.

The optical sectioning strength for each corresponding illumination frequency (from Table 1) was measured using the phantoms and methods described previously and also depicted in Figure 2. The optical sectioning thickness decreased as the illumination spatial frequency increased, as expected. The results detailing the experimental relationship between optical section thickness and illumination frequency are displayed in Figure 4. The plot on the left shows a single axial scan through one phantom (single layer of 1 µm spheres, µ_s_′ = 0 cm^−1^) using an illumination frequency of 19.6 mm^−1^. The optical section thickness was defined as the distance at which the mean intensity dropped to 50% of the intensity at the focal plane. The plot on the right shows the measured optical section thickness in all three phantoms (each with a different scattering level) at all available illumination frequencies. Each of the three phantoms is represented with a different symbol. Overall these results clearly show a non-linear relationship between optical section thickness and illumination frequency. Error bars on each measurement were generated by selecting eight different regions of interest within each image stack and measuring the standard deviation among the optical sectioning thickness of all regions. Statistical analysis of the data using a Student’s t-test demonstrated no significant difference between scattering levels. The measured optical section thickness data was compared to a theoretical value acquired using the Stokseth approximation to the optical transfer function (Eq. 4) described in the Methods section. As shown in Figure 4(A) the measured data of the single axial scan matched with Stokseth approximation (using the variables *ν* = 19.6 mm^−1^, *λ* = 520 nm, and NA = 0.1). Also shown in Figure 4(B), the measured optical section thicknesses showed excellent agreement with the theoretical optical section thickness calculated using the Stokseth approximation.

**Figure 5:**
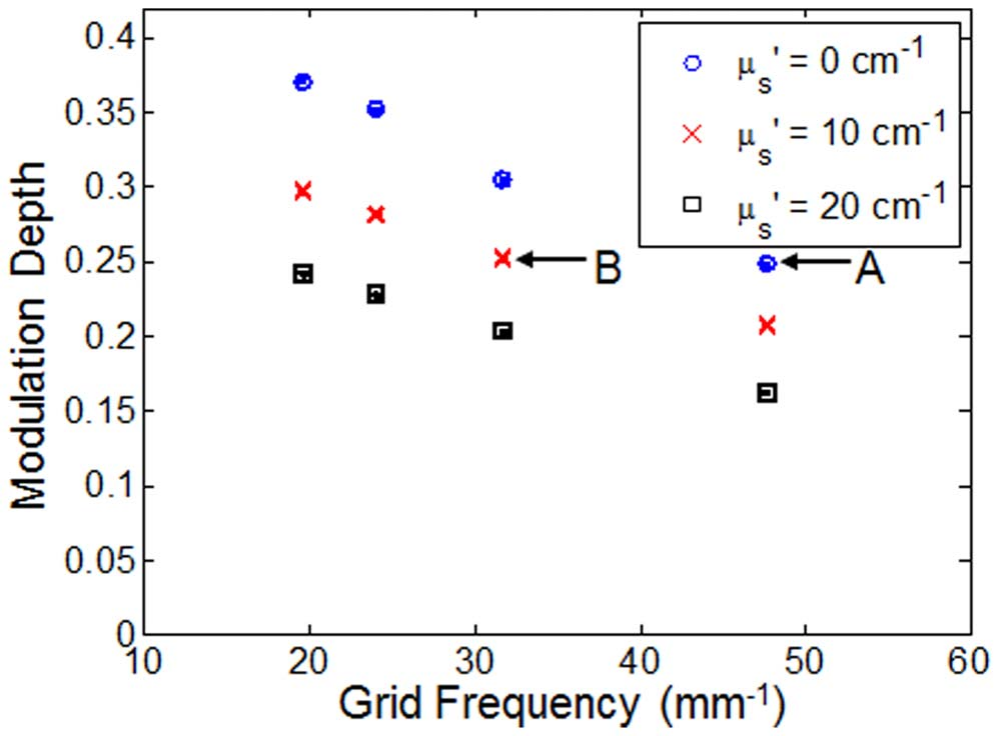
Modulation depth as a function of grid frequency for non-scattering phantoms and scattering phantoms (two µ_s_′ levels). The points labeled A and B are referenced in the Discussion section.

After determining the frequency dependence of optical section thickness, the modulation depth at each illumination frequency was measured using the method described by Hagen et al. [32]. The measured modulation depths in two separate phantoms (µ_s_′ = 0 and 10 cm^−1^) for each frequency are shown in Figure 5. The modulation depth was calculated, as described in the Methods section, over an identical ROI in each image for all illumination frequencies using the same laser illumination power and CCD integration time. Error bars were generated by computing the standard deviation of modulation depth over 10 images at the same sample location. The plot clearly indicates an inverse relationship between modulation depth and illumination frequency. In addition, a significant decrease in modulation depth at all frequencies is seen in the phantom with µ_s_′ = 10 cm^−1^ compared to the non-scattering phantom.

**Figure 6:**
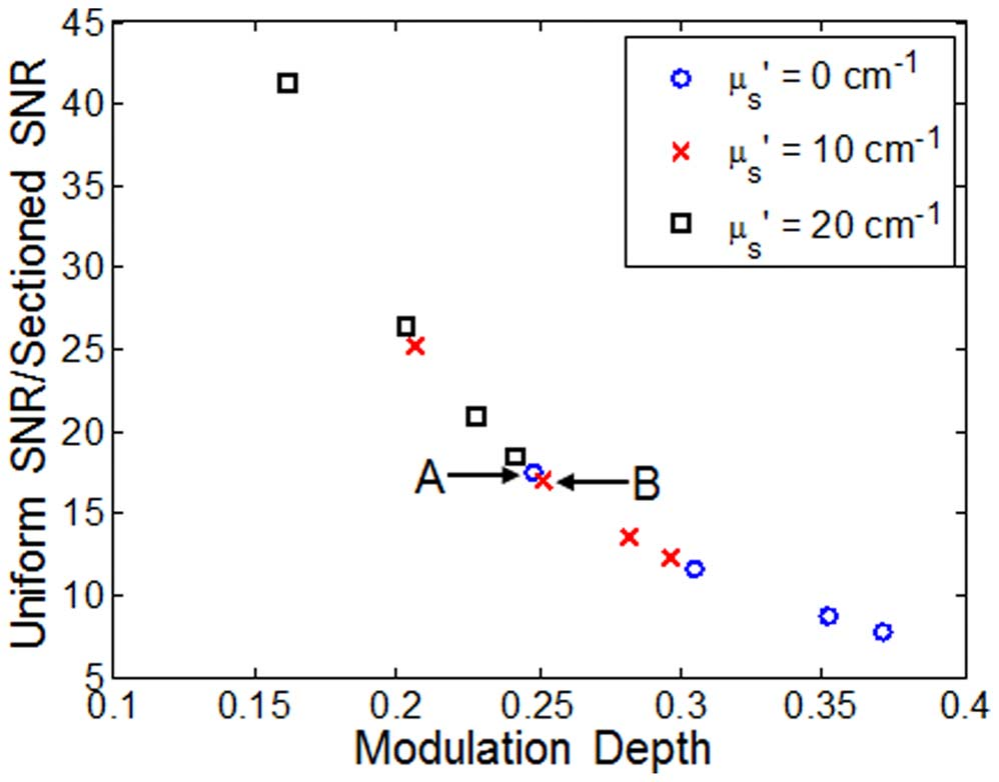
SNR ratio (uniform SNR to sectioned SNR) versus modulation depth for non-scattering and scattering phantoms. The points labeled A and B are referenced in the Discussion section.

Figure 6 shows a plot of the ratio of uniform SNR to sectioned SNR against modulation depth (i.e., it represents the reduction in SNR of SIM compared to standard widefield microscopy). As the data demonstrates, the ratio decreases with modulation depth, indicating less degradation in SNR at higher modulation depths compared to lower modulation depths.

### Demonstration of System in a Mouse Model of Sarcoma

**Figure 7:**
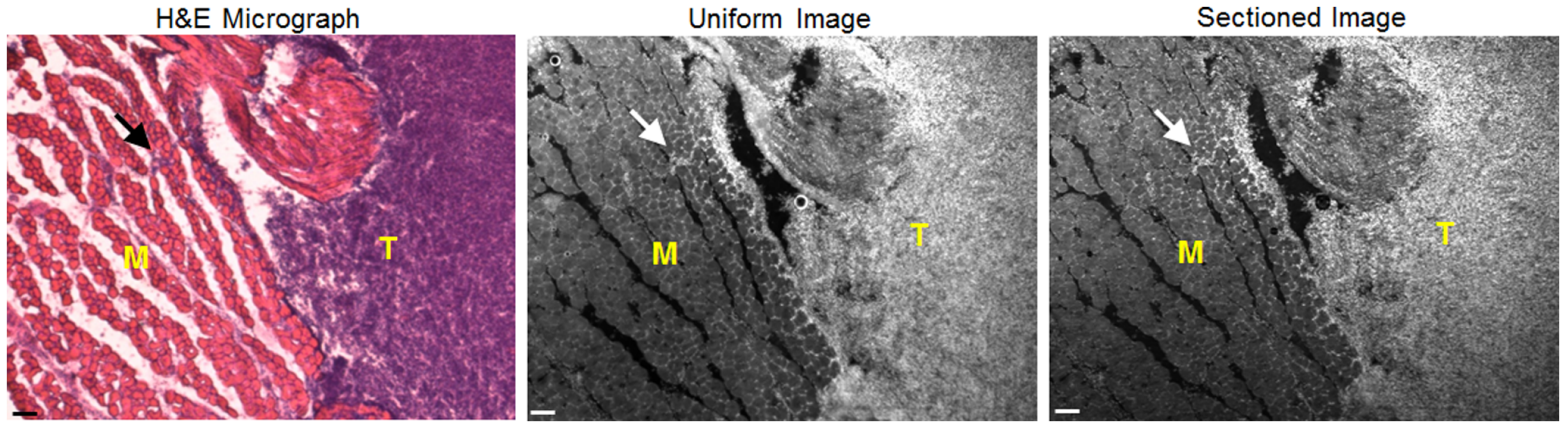
Images of a 50 µm tissue slice demonstrating the correlation between images acquired by the SIM system and H&E histological micrograph. SIM images were taking using the 4×objective and a frequency of 31.7 mm^−1^. H&E images were taken using a 2.5×objective and the images were cropped to match one another. Site imaged on tissue contains both cross sectional skeletal muscle (M) and sarcoma tumor (T). The arrows on each image point out a site where tumor tissue was invading into the normal skeletal muscle. Scale bars are 100 µm.

To demonstrate feasibility of imaging tissue histology with appropriate resolution, the system was tested on 50 µm thick frozen tissue sections cut from a sarcoma tumor harvested from a primary mouse model of sarcoma. Representative images are shown in Figure 7. These images demonstrate excellent congruence between the morphological images acquired by the SIM system and the H&E micrographs. It is clear from these images that the AO stains both the cell nuclei and skeletal muscle. Importantly, the features of the H&E image are recapitulated in the AO image based on intensity differences: the cell nuclei are more brightly stained than the muscle tissue. The sarcoma tissue can be observed invading into the muscle tissue in both the H&E and AO image. Because these were thin tissue sections, the images did not contain a large amount of background fluorescence. As can be seen, there is not substantial image contrast improvement from the uniform to the sectioned image (acquired at f = 31.7 mm^−1^).

**Figure 8:**
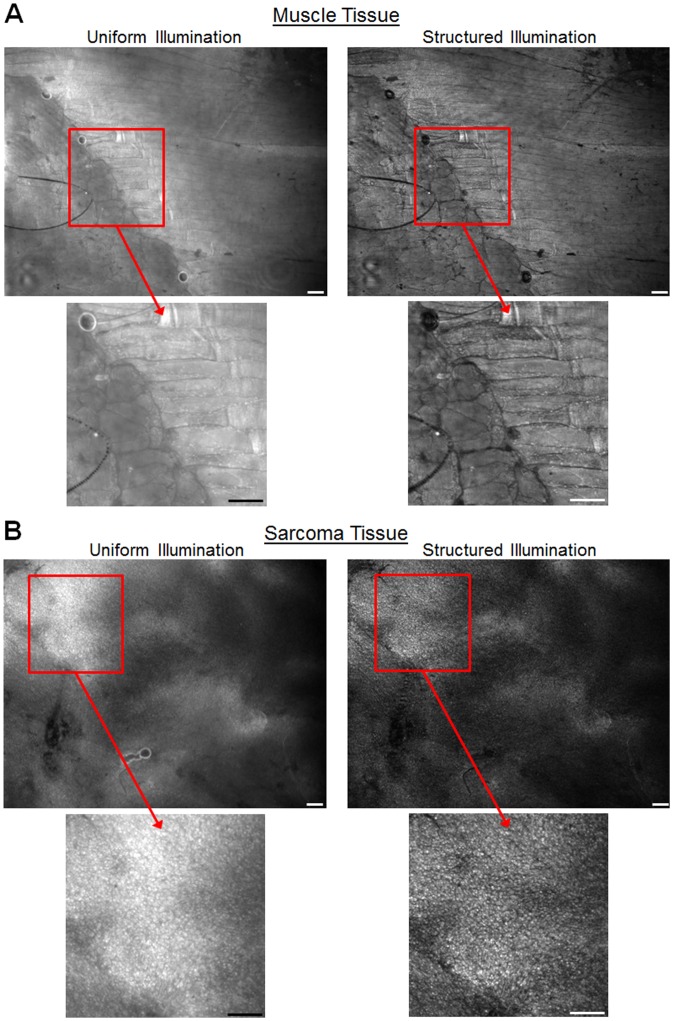
Uniform and structured illumination images acquired from mouse tissue. (A) Images of skeletal muscle from mouse. Both longitudinal and cross-sectional muscle can be seen in the region of interest (B) Image of tumor tissue from mouse sarcoma. Cell nuclei are the only source of contrast apparent in these images. Contrast enhancement is clearly seen in the sectioned images (acquired at f = 31.7 mm^−1^). Scale bars are 100 µm.

To demonstrate the feasibility of imaging of thick tissue, freshly excised sarcoma tumor tissue was imaged using the SIM system. Representative images (uniform and sectioned) of mouse skeletal muscle are shown in Figure 8(A) and sarcoma tumor in Figure 8(B). Because these tissue samples are intact and non-sectioned a large amount of scattered background fluorescence is visible in the uniform illumination images. As a result, the contrast enhancement provided by structured illumination improves visualization of individual muscle fibers in normal tissue and individual cell nuclei in tumor tissue. Both of these tissue sites were imaged with a frequency of 37.1 mm^−1^, which resulted in median modulation depths of 0.24 and 0.17 in the muscle and tumor, respectively.

### Analysis of Images using a HPF Algorithm for Quantification of Nuclear Density

As shown in Figure 8, application of AO as a fluorescence contrast agent enabled the SIM system to clearly visualize cell nuclei in both frozen sections and intact tissue. While qualitative differences were seen in these images, a quantitative metric such as nuclear density would aid in assessing the effect of illumination frequency on the quality of the sectioned image. As an initial approach, a simple HPF algorithm was used to isolate the cell nuclei described in the methods section. This was first applied to a set of eight images acquired from frozen sections of a sarcoma tumor from four different mice (two images per mouse). The samples were specifically selected so that pure sarcoma tumor tissue (confirmed by H&E staining) was visible and an ROI (350×350 pixels) containing only tumor tissue was manually selected from each image. Quantitative analysis of this image set indicated that the average cell nuclei count was 3561±754 nuclei/mm^2^ over a single ROI of pure sarcoma tumor tissue averaged across the eight images (mean ± standard deviation calculated from different FOVs of sarcoma tissue harvested from one animal).

**Figure 9:**
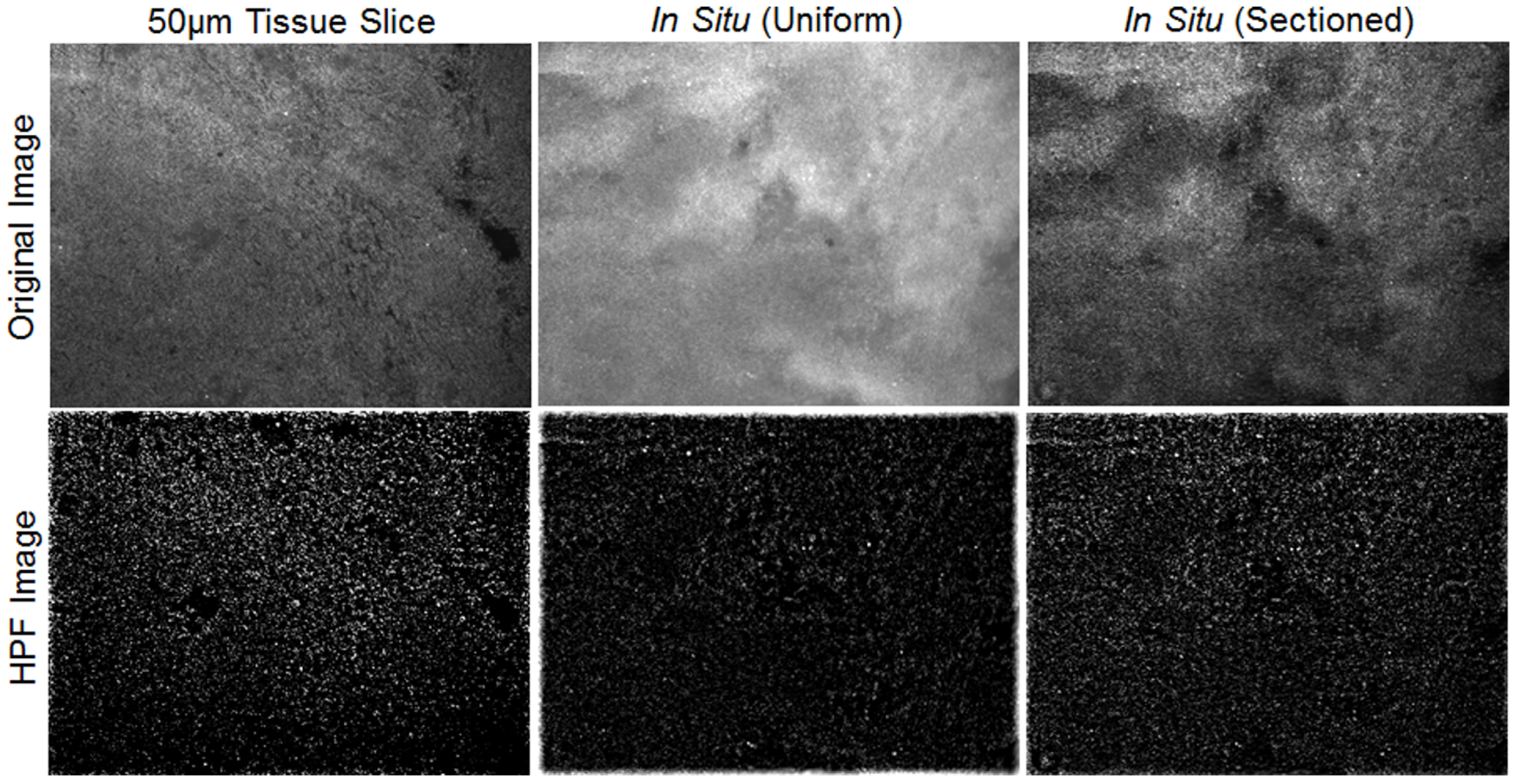
Fluorescent images of mouse sarcoma tissue processed using a high-pass filter (HPF) algorithm to isolate cell nuclei. The *in situ* sectioned image (acquired using f = 31.7 mm^−1^) closely resembles the 50 µm tissue slice image (acquired from frozen sections), while the *in situ* uniform image failed to isolate the majority of cell nuclei.

Next, the HPF quantification algorithm was applied to images of thick *in situ* sarcoma tumor to determine the impact of different illumination frequencies on the quantitation of nuclear density. Again, the sample was selected to ensure that tumor tissue was imaged and the same ROI (350×350 pixels) was imaged using three different illumination frequencies (24.1, 31.7, 47.7 mm^−1^). The original frozen section (50 µm tissue slice), *in situ* uniform, and *in situ* sectioned images (only f = 31.7 mm^−1^ shown) and corresponding HPF images are displayed in Figure 9. These images demonstrated that applying HPF to the *in situ* uniform image was unable to accurately isolate cell nuclei, while also applying HPF to the *in situ* sectioned image isolated more cell nuclei, which closely resembled the 50 µm tissue slice.

**Figure 10:**
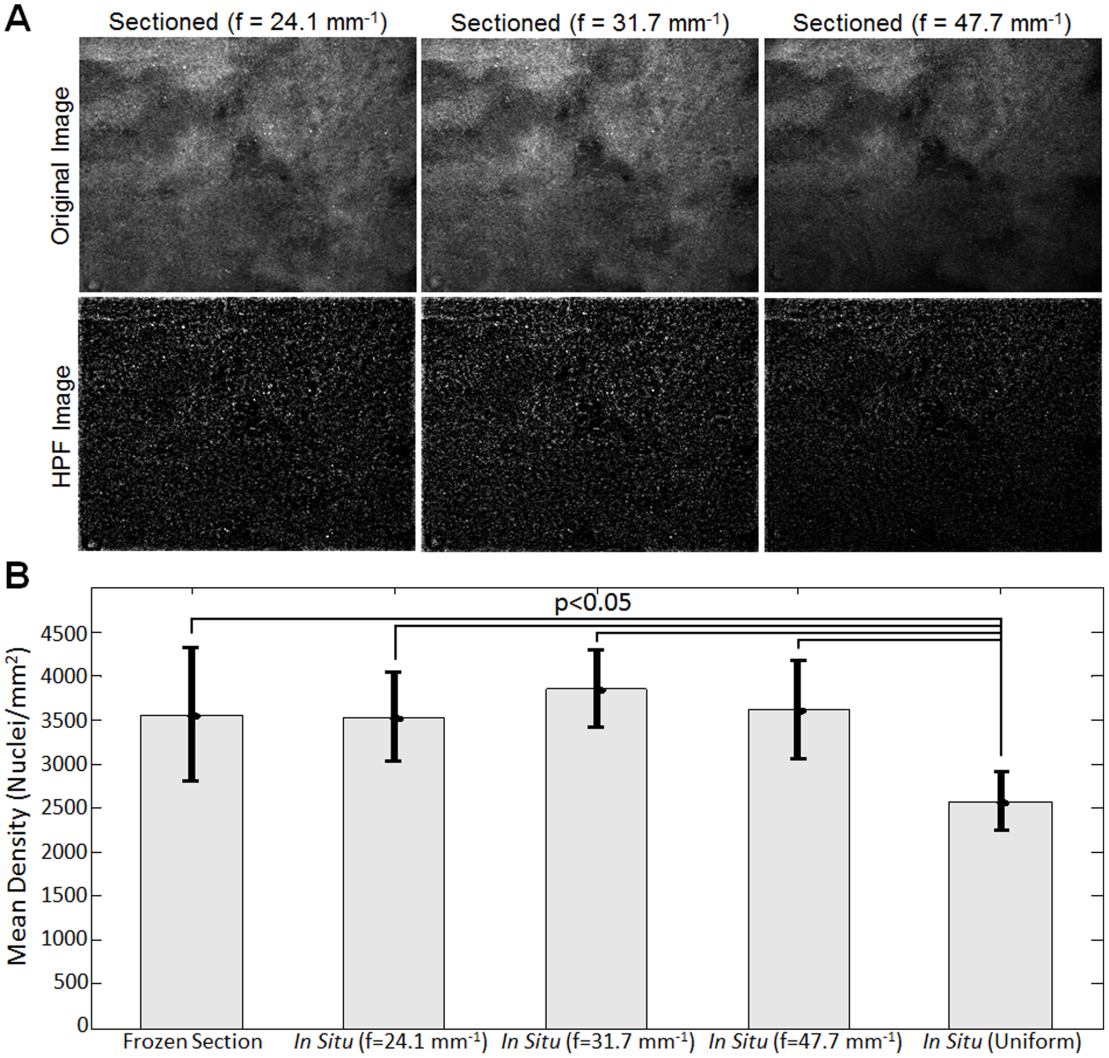
Results demonstrating the impact of illumination frequency on quantification of cell nuclei density. (A) Sectioned images of mouse sarcoma tissue at multiple illumination frequencies. The corresponding HPF processed images are shown which demonstrate the isolation of cell nuclei. (B) Mean density of cell nuclei extracted from images mouse sarcoma tissue. The nuclear density extracted from the 50 µm thick frozen section images (N = 9) was compared to density extracted from *in situ* tissue imaging (N = 5), both uniform and sectioned (f = 47.7, 31.7, 24.1 mm^−1^) images.

The corresponding sectioned images of sarcoma tissue at each illumination frequency are shown in Figure 10(A), which qualitatively show the impact of frequency on isolating cell nuclei. For a quantitative analysis, the mean nuclear density was calculated on a set of five separate images of *in situ* sarcoma tumor tissue (different FOVs of sarcoma tissue harvested from one animal). This was done on both the uniform illumination and sectioned images acquired at three illumination frequencies and a comparison to the mean nuclear density from the 50 µm frozen section images are shown in Figure 10. The p-values were calculated using a Wilcoxon rank-sum test, and revealed that the *in situ* density from the uniform illumination images were significantly lower (p<0.05) than the quantified density from the 50 µm tissue slice images and the sectioned images at all three frequencies. Specifically, the nuclear density from the uniform illumination images was smaller than the density in the tissue slice and sectioned images indicating that the lower contrast between nuclei and the background seen in the uniform illumination (i.e., non-sectioned) images leads to underestimation of nuclear density.

## Discussion

Structured illumination is an elegant approach to solve the problem of optical sectioning in microscopy, essentially analogous to frequency modulation techniques used to encode electrical signals. Structured illumination is a low-complexity solution for optical sectioning microscopy of thick tissues that has the potential for clinically feasible high throughput microscopy of tumor margins due to its light efficiency and parallel-pixel detection approach. We have presented a custom SIM microscopy system with a low magnification, low NA objective designed to maximize the single-frame FOV for applications requiring large area tissue surveillance such as is the case for tumor margin imaging. We designed a series of experiments to fully characterize SIM performance for tumor margin imaging, and demonstrated that SIM (optical sectioning) of thick tissue provides equivalent quantitation of nuclear density, a hallmark of carcinogenesis, as compared to frozen section analysis (physical sectioning) using the same fluorescent staining approach.

The choice to use an SLM to generate the structured pattern for illumination allowed us to explore the role of frequency in optical sectioning performance in thick, turbid samples. It is generally understood that using a higher illumination frequency yields a thinner optical section at the focal plane, leading to greater background rejection and enhanced contrast at the focal plane. While this is a desirable result, the caveat of using a higher frequency is a decrease in modulation depth primarily due to the attenuation of higher pattern frequencies by the optical transfer function of the illumination optics. Furthermore, the effect of sample turbidity on optical sectioning performance with SIM had not been systematically investigated prior to this work. As demonstrated in Figure 4, regardless of the underlying scattering properties of a sample, increasing the illumination frequency yields increasingly thinner optical sections (which follow theoretical calculations of attenuation of signal from a thin fluorescent sheet with defocus). However, the decrease in optical section thickness is not linear with respect to illumination frequency and actually follows an asymptotic relationship. It has been previously shown that the thinnest optical section that can be achieved is using a normalized grid frequency 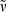 (ref. Eq 6) equal to 1 [42]. Given the specific parameters of our system (NA = 0.1, *λ* = 520 nm), this would correspond to an absolute frequency of 191 mm^−1^, resulting in an optical section thickness of 36.6 µm. Using the Stokseth approximation, 90% of the thinnest optical section (40.6 µm) is achieved at a frequency of 132 mm^−1^. An additional 10% increase of the optical section thickness to 45.7 µm occurs at an illumination frequency of 106 mm^−1^. In other words, increasing the illumination frequency from 106 mm^−1^ to 191 mm^−1^ (an 80% increase in illumination frequency) only results in a 20% decrease (<10 µm) in optical section thickness. From this data, it is clear that there are diminishing returns when trying to achieve a thinner optical section by increasing the frequency.

In contrast to the optical section thickness, the modulation depth decreases nearly linearly with increasing spatial frequency as shown in Figure 4. This is important because the SNR is directly linked to modulation depth and as Figure 5 demonstrates, this relationship is also non-linear. This indicates that not only is there potentially less benefit in increasing the frequency to reduce optical section thickness as the frequency cutoff of the optical transfer function is reached, but in doing so it would also significantly degrade the SNR of the sectioned image as well. For example, looking specifically at the illumination frequency of 31.7 mm^−1^, the corresponding optical section thickness at this frequency was 129 µm. If the illumination frequency is increased to 47.7 mm^−1^, then the expected optical section thickness would be decreased by 29%. However, the SNR would be disproportionally decreased by 217%, assuming that the reduced scattering coefficient of the medium, µ_s_′ is 10 cm^−1^.

Figure 5 indicates that the achievable modulation depth is also affected by the amount of background signal, which underscores the need to measure modulation depth in the target tissue before choosing the optimum illumination frequency. Additionally, Figure 6 shows that with knowledge of modulation depth, the SNR reduction in sectioned images can be estimated. A comparison between two specific data points in Figure 6, referred to as point A (illumination frequency = 47.7 mm^−1^, µ_s_′ = 0 cm^−1^) and point B (illumination frequency = 31.7 mm^−1^, µ_s_′ = 10 cm^−1^), is instructive. While they have noticeably different illumination frequencies and scattering properties, Figure 5 shows that the measured modulation depth of points A and B are actually very similar, 0.248 and 0.252, respectively (∼2% difference). And it follows, as no surprise, that in Figure 6, the uniform SNR to sectioned SNR ratio for point A and B are 17.5 and 17.1, respectively, which are also very similar (∼2% difference). Regardless of how a particular modulation depth is achieved, either through using a certain illumination frequency, altered by scattering background, or the combination of the two, the SNR reduction is only dependent on the actual measured modulation depth.

Finally, the images from pure sarcoma tumor tissue, which were processed using the HPF algorithm and shown in Figure 9, demonstrated the value of SIM, as standard uniform illumination failed to isolate the majority of cell nuclei and significantly underestimated the nuclear density (shown in Figure 10). Interestingly, the statistical analysis also showed that regardless of the three illumination frequency selected, the quantified nuclear densities from the optically sectioned images were not statistically different from the 50 µm tissue slices. As a compromise between optical section thickness and SNR reduction, the illumination frequency of 31.7 mm^−1^ was selected for this specific system and imaging application. At this frequency, the expected optical section thickness was 129 µm and the median modulation depth over the entire image was 0.137. Although this modulation depth corresponded to a 50-fold reduction in SNR from the widefield case, it was clear that this was outweighed by the improved contrast with respect to not only visualizing, but also properly segmenting tumor cell nuclei. It is worthwhile to note that this combination of illumination frequency, wavelength, and NA yield a normalized frequency (introduced in Eq.4) of 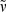 = 0.165. Previous studies have shown that a normalized frequency of 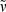 = 1 yields the thinnest possible optical section [31], [42]. While this certainly holds true in the case of our system, we determined that use of the lower spatial frequency provided sufficient benefit from background rejection due to optical sectioning, while still retaining enough signal to identify critical structural information in the sectioned images. Future controlled studies on a larger cohort of animals will determine the accuracy of using this system to differentiate between positive and negative tumor margins.

The conclusion for illumination frequency choice was intended specifically for our system and biological application described in this paper. In other applications and system configurations, the optimal illumination frequency may be different than the one chosen. We note here that although we used a 4×objective in this implementation to achieve a desired balance between single-frame field of view and resolution, that the resolution could be increased at the expense of single-frame field of view, by going to higher power objectives. Mosaics can be constructed which provide an overall larger field of view, and allow the images to be viewed digitally at a range of magnifications (larger than the single-frame magnification). However, our results demonstrated that the optical section thickness for a given microscope objective could be accurately represented using the Stokseth approximation regardless of the scattering properties of the tissue. Additionally, the SNR reduction in the sectioned image was only dependent on modulation depth and independent of tissue scattering. While the scattering did affect the modulation depth, it can be measured and quantified directly without knowing the exact scattering properties of the tissue. Therefore, optical section thickness and SNR reduction can be calculated simply by knowing the objective NA and measuring the modulation depth, respectively. This yields a straightforward approach for one to characterize structured illumination and determine the appropriate illumination frequency for a specific application.
